# Marital dissolutions and changes in mental health: Evidence from rural Malawi

**DOI:** 10.4054/demres.2021.44.41

**Published:** 2021-05-12

**Authors:** Tyler W. Myroniuk, Hans-Peter Kohler, Iliana V. Kohler

**Affiliations:** 1 University of Missouri, Department of Public Health, 825 Lewis Hall, 65211 Columbia, MO, USA.; 2 Population Aging Research Center (PARC) and Department of Sociology, University of Pennsylvania., USA.; 3 Population Studies Center (PSC) and Department of Sociology, University of Pennsylvania, USA.

## Abstract

**BACKGROUND:**

Advancing efforts to unpack the complex relationship between marital dissolutions and health outcomes increasingly requires assessing the marital histories and health of individuals who have lived long enough to experience divorce or widowhood ‒ or even multiples of each ‒ and measurable changes in health.

**OBJECTIVE:**

To explore this line of inquiry, we chose a sample from rural Malawi where a high prevalence of marital dissolutions and remarrying exists, as an ideal theoretical foil to the predominant literature found in high-income countries (HICs). We examine if changes in having experienced a marital dissolution, one’s total number of dissolutions, and the percentage of one’s life spent outside of marriage since first becoming married are associated with changes in mental health.

**METHODS:**

Our analyses focus on 1,266 respondents aged 45 years and older who participated in the 2012 Mature Adults Cohort of the Malawi Longitudinal Study of Families and Health (MLSFH-MAC), linked back to cohort information from 2008 and 2010 available through the MLSFH. Fixed-effects regressions guide our inferences over the 2008, 2010, and 2012 waves of data.

**RESULTS:**

For men, spending more life outside of marriage is associated with worse mental health, while more marital dissolutions are surprisingly associated with better mental health for women.

**CONCLUSIONS:**

These results could suggest that larger portions of one’s life spent unmarried are associated with a type of role strain for men or simply that men are burdened with taking up tasks that their spouses had previously done in order to survive. For women, many may have gotten out of ‘bad’ marriages that otherwise would have been detrimental to their mental health and/or those in good mental health are the ones able to remarry.

**CONTRIBUTIONS:**

Our research from rural Malawi provides a type of litmus test for many HICs where marriage, remarriage, and dissolution rates are lower but quite consequential for mental health outcomes. Measuring time outside of marriage should be more strongly considered in such settings. These results also inform increasingly important research on the relationship between marital dissolutions and mental health in other African nations as noncommunicable diseases play a continually more important role in people’s lives.

## 1. Background

Advancing efforts to unpack the complex relationship between marital dissolutions and health outcomes increasingly requires assessing the marital histories and health of individuals who have lived long enough to experience divorce or widowhood ‒ or even multiples of each ‒ and measurable changes in health. The discrete events of divorce or widowhood have been researched most heavily, and unsurprisingly, they are associated with worse mental and physical health in both the short- and long-term (e.g., Blekesaune 2008; Goldman, Korenman, and Weinstein 1995; Lillard and Panis 1996; Smith and Zick 1996; Stroebe, Schut, and Stroebe 2007; Strohschein et al. 2005; Waldron, Hughes, and Brooks 1996). Emotional strains and the loss of crucial resources are often cited as mechanisms linking divorce and/or widowhood to worse health outcomes in the aftermath (Carr 2004; Elwert and Christakis 2006; Pearlin 1989; Pearlin 1999; Sansom and Farnill 1997; Tavares and Aassve 2013). The accumulation of time spent in and out of marriage matters for health, too (e.g., Amato and Previti 2003), but has not been as readily pursued as the counting of discrete marital events. For example, the duration between divorces, widowhood, and marriage is strongly connected to worse health and greater risk of the onset of disease for men (Dupre and Meadows 2007; Dupre, Beck, and Meadows 2009). Also, the health protective effects associated with prior marriages last longer for men than women, but men have a much higher risk of mortality between marriages (Brockmann and Klein 2004). Of course, health selectivity adds to the difficulty of unpacking these relationships (see Rendall et al. 2011).

## 2. Objective

There is an abundance of extant research on the relationship between marital dissolutions and health set in high-income countries (HICs) where the incidence of divorce and remarriage have long been increasing but entry into marital unions is also declining (Cherlin 2012; Lesthaeghe 2010; Sobotka 2008; van de Kaa 1987). A change of scenery could provide new insights in advancing our understanding of the sheer complexity in this relationship; a context where marriage is universal and marital transitions are highly prevalent would seemingly be ideal. For the following reasons, we believe that Malawi, a low-income country in sub-Saharan Africa (SSA), is actually a well-suited place to advance this line of theoretical inquiry.

First, over 98% of individuals aged 45 years and older in rural Malawi have been married (authors’ calculations; Malawi National Statistical Office 2011), and the median age at first marriage is early (roughly 17 to 18 years for women, 22 to 23 years for men; Malawi National Statistical Office and ICF Macro 2011). Effectively, everyone is eligible for a marital dissolution ‒ whether through divorce or widowhood ‒ and from a young age.

Second, divorce rates have historically been high in Malawi, or at least as long as researchers have examined marriage (e.g., Mitchell 1951; 1956). An estimated 28% to 49% of marriages end in divorce before a couple’s twentieth anniversary (Bracher, Santow, and Watkins 2003).

Third, Malawians have a high, expedient incidence of remarriage after marital dissolutions; remarriage is often pursued because of the key economic role of the marital household and social expectations to be married (Reniers 2003).

Fourth, while divorce is normative, successive generations tend to willfully ignore its prevalence (Kaler 2001), which allows marriage to remain socially important.

In short, marriage in Malawi is nearly universal with significant levels of marital instability and marriage turnover. We test how changes in marital status, the number of marital dissolutions, and time spent outside of marriage are associated with changes in rural Malawians’ mental health. We pursue our research through a longitudinal sample of mature adults, defined as individuals aged 45 and older, in rural Malawi, where roughly 85% of Malawi’s population of roughly 17 million (UNDP 2015) lived when data were collected (Malawi National Statistical Office 2011).

Changes in mental health are important to consider here, in part, because they precede declines in physical health outcomes of rural Malawians (Kohler et al. 2017). These changes are highly gendered too: Older Malawian women, in general, have worse mental health outcomes than men (Kohler et al. 2017), but men who are not married have among the worst mental health outcomes of any other demographic group (Clark et al. 2020).

Not to forget, individuals 45 years and older are those who survived the worst of Malawi’s HIV/AIDS epidemic. The stresses of being in a context where one has to worry about becoming infected with HIV has been detrimental to rural Malawians’ mental health (Hsieh 2013), including being an important driver of accumulated bouts of depression and anxiety contributing to declines in mental health among older Malawians (Kohler et al. 2017). On top of this, due to the health resources going overwhelmingly toward HIV/AIDS prevention and treatment, even the Malawi Ministry of Health recognizes that “general health care workers have no competency for handling mental health conditions” (Udedi 2016).

Malawi can be informative in this larger, global question of the relationship between marital dissolutions and health. If there are health consequences to individuals who spend lots of time outside of a marital union, in a place where marriage is normative, divorce is normative, widowhood is inevitable (because widowhood is experienced only via marriage), and there are strong social and economic incentives to be married, then Malawi offers an ideal theoretical foil to HICs and other SSA settings; these places are where the quantum and tempo of marital transitions are not as high as Malawi, and time outside of marriage could be just as, if not more, consequential.

## 3. Context: Marital dissolutions and strategy in Malawi

As noted above, being married at least once is almost universal in rural Malawi (Malawi National Statistical Office 2011). However, the contemporary importance of marriage, as an institution in Malawi, has rarely been systematically documented in the last two decades (two notable exceptions are Kaler 2001 and Wilson 2008) and nearly a half century prior to that (i.e., Mitchell 1951; Mitchell 1956).

Women enter into marriages fully expecting to be unable to trust their husbands and ‒ in most of Malawi ‒ in patrilineal, and potentially hostile, settings (Wilson 2008). In the rest of Malawi ‒ matrilineal Malawi ‒ women can fairly easily dismiss their husbands: “Take your mat and go” (Schatz 2005). With high ‒ or perceived high ‒ risks of HIV infection, divorce has been postulated as a mechanism in which to avoid widowhood and becoming infected; long spells of being in an unmarried state then can lead to suspicion of a spouse having died of HIV/AIDS (Watkins 2004). Information asymmetry about individuals’ HIV statuses and fidelity lay the groundwork for this widespread strategy among rural Malawians (see Angelucci and Bennett 2017). In many ways, divorce can be used to avoid widowhood ‒ rendering them ‘two sides of the same coin.’ Therefore, it is not shocking that mature adults are highly likely to have experienced a divorce (Bracher, Santow, and Watkins 2003; Reniers 2003).

Further, in rural Malawi, individuals who experience a marital dissolution believe that they have higher chances of dying in the coming years compared to married individuals (Delavande and Kohler 2009). Also, those who have been divorced or widowed for longer than two years have worse mental health outcomes than those who experienced more recent marital dissolutions (Myroniuk 2017). Rural Malawians have strong health incentives to remarry, and quickly, after dissolutions.

Gendered differences in experiences of marital dissolutions also factor into individuals’ strategies. Women who experience a marital dissolution are at a disadvantage in terms of partner selection when attempting to reenter the marriage market in Malawi for most reasons imaginable ‒ such as health, education, age, and other characteristics (Anglewicz and Reniers 2014; Reniers 2008). Yet, some factors might buffer these negative consequences of marital dissolutions. For instance, in other SSA settings, savings are key to the health and well-being of unmarried individuals, especially for older women (e.g., Cliggett 2005; Ingstad et al. 1992). In matrilineal kinship systems ‒ which are predominant in Malawi’s southern region ‒ men generally do not get access to their deceased or divorced wives’ land rights and must return to their home village, which might hint that marital dissolutions could be less detrimental to women’s health in matrilineal, as compared to patrilineal, contexts. For example, in patrilineal settings (mainly in Malawi’s central and northern regions), divorcées must return their bride-wealth payment and go back to their family’s village. In contrast, widowed women can retain their husbands’ inheritance rights (see Takane 2008; Berge et al. 2014). It is critical to understand whether these factors, like household savings or region of residence, mitigate the possible negative health consequences of marital dissolutions, and whether there are, indeed, particular consequences by gender. Coupled with gendered disparities in remarriage markets ‒ specifically, older divorced and/or widowed women needing to remarry in order to survive financially ‒ divorce and widowhood operate in similar ways for women.

## 4. Guiding research questions

By exploiting data from the Mature Adults Cohort of the Malawi Longitudinal Study of Families and Health (MLSFH-MAC; Kohler et al. 2020), we have two research questions:

1. Are experiencing a marital dissolution, increasing one’s total number of dissolutions, and spending a greater percentage of one’s life spent outside of marriage since first becoming married associated with rural Malawians’ changes in mental health?
  The former two measures of marital dissolutions are conventional, whereas the latter measure is a new, complementary measure that takes into account the potential negative social and economic consequences of the amount of time one spends out of marriage in a setting where one is expected to be married.
2. Do household life savings and region of residence ‒ both of which have consequences for women and men in Malawi ‒ moderate the relationship between these aspects of marital dissolutions and mental health?
  This has been noted as an understudied component of older individuals’ livelihoods, even in HICs (Bowen and Jensen 2017).

## 5. Data and methods

### 5.1 Study details

Our analyses use data from the Mature Adults Cohort of the Malawi Longitudinal Study of Families and Health (MLSFH-MAC). MLSFH-MAC is a population-based cohort study of mature adults, aged 45 years and older, living in rural communities in three districts in Malawi (Balaka in the southern region, Mchinji in the central region, and Rumphi in the northern region). MLSFH-MAC was derived from the Malawi Longitudinal Study of Families and Health (MLSFH), an ongoing longitudinal panel study established in 1998 and one of few long-standing, publicly available longitudinal cohort studies in SSA with up to 4,000 individuals. The MLSFH documents more than two decades of demographic, socioeconomic, and health conditions in one of the world’s poorest countries. MLSFH cohorts (and a result, MLSFH-MAC) were selected to represent Malawi’s substantial rural population (“Cohort Profiles” of the MLSFH-MAC and MLSFH, providing detailed discussion of sampling procedures, survey methods, survey instruments, and analyses of attrition, which have been published and/or are available as a working paper [Kohler et al. 2015; Kohler et al. 2020]). Importantly, attrition does not appear to bias the estimation of key health-related relationships in the MLSFH data (Kohler et al. 2015).

Our analyses center on the MLSFH-MAC respondents who were 45 years and older in the 2012 survey (N = 1,266) that focused on mental health and well-being among older adults who had previously been interviewed in the 2008 and 2010 MLSFH. Thus, the sample consists of individuals who were 45 years and older in the 2012 survey and had participated in the prior two waves of data collection; this choice is not only practical for evaluating change in our marital and health variables but also a key part of the MLSFH-MAC research design. For those eligible to be in the mature adult sample in 2012 (and thus participated in 2008 and 2010), slightly under 3% died by 2012. Since few eligible individuals for these mature adult analyses died in between waves, bias due to mortality in our analyses is minimized (Kohler et al. 2020).

Refusal rates were low and those who took part in the 2008 and 2010 waves but were not found in 2012 either died or migrated (for example, of the eligible individuals for our sample who were alive in 2008, slightly more than 3% died by 2010). Accounting for irreconcilable marital histories based on 2012 data collection (N = 64, described below in greater detail), and age being recorded as less than 45 in 2012 (N = 16), our analytic sample includes 1,186 adults aged 45 and over in 2012 (670 women, 516 men) who participated in the 2008 and 2010 waves of the MLSFH as well. About 10% (N = 171) of the 2012 mature adult sample is excluded due to missing values on age at first marriage or implausible values, which accounts for differences in observations in some regression models.^4^ The empirical consequences of these missing data are outlined and dealt with later in the paper, including Appendix Table A-1, which is a complete case regression analysis and discussion of multiple imputation results (not presented).

### 5.2 Variables

Our dependent variable, the SF-12 mental health scale ranges from 0 to 100 (from worst to best mental health). The twelve-item, self-rated survey measure has been widely validated as a holistic depiction of individual mental health (Gandek et al. 1998; Ware, Keller, and Kosinski 1995) and is strongly correlated with other depression and anxiety measures in the MLSFH (Kohler et al. 2017).^5^ Recently, the SF-12 has been validated in rural Malawi, with the SF-12 mental health scale capturing other dimensions of mental health even better than in US populations (Ohrnberger et al. 2020). For the SF-12 measure, which was collected for individuals over all three waves of the data in this study, about 40% of women and men have at least one SF-12 score that differs by one standard deviation from the mean. Considering that two standard deviations below the SF-12 mental health scale mean indicates clinical depression (Ware, Keller, and Kosinski 1995), while a score of 45 or less ‒ about one standard deviation below the mean score for women and men in our sample ‒ has been used as a general cutoff for depression screening (Gill et al. 2007), it appears that there is sufficient variation in the SF-12 within individuals, across waves, to capture meaningful changes in mental health. Ideally, mental health would be measured with greater frequency via more waves of data, but this empirical problem is common even in well-established HIC survey research. We also acknowledge that there are important baseline mental health disparities between respondents, but these potential sources of bias in our analyses are ameliorated, to a large extent, by the mechanics of fixed-effects regressions that cancel out these by estimating changes in health only within individuals.

The availability of detailed marital histories for MLSFH respondents are essential for the present analyses. Although marital histories among MLSFH participants are not always consistent with one another across waves (Chae 2016), we address this issue, as much as possible, by starting with data from the 2010 marriage history roster for individuals who participated in the 2008, 2010, and 2012 waves. The 2010 roster captures 2008 and 2010 marriage histories. The 2012 wave explicitly recorded updates to one’s marriage history since 2010 (linked to spousal data from 2010), and these changes are reflected in the 2012 measure of marital status in our analyses; the 2012 marriage section of the questionnaire was conducted differently than in 2012, resulting in 64 marital histories that we could not reconcile with 2010 and had to drop to ensure conservative estimates.^6^ Although imperfect, this method allows for internal consistency in measuring marital status.

To more effectively test how changes in our three primary independent variables ‒ ever divorced/widowed, number of marital dissolutions, and the percentage of one’s life spent divorced and/or widowed (thus outside of marriage) since first being married ‒ predict changes in older Malawians’ mental health, we utilize fixed-effects regression analyses, combining health outcomes from the 2008, 2010, and 2012 waves and time-varying marital indicators. For conceptual reasons, noted above, we do not distinguish between divorce and widowhood but rather combine these as being in an unmarried state.

Our newly proposed continuous measure innovatively allows us to aggregate complicated marital histories and potentially capture nuance in the social consequences of spending more time of one’s life outside of marriage, compared to the other two discrete measures. It was calculated from the MLSFH’s marriage history roster whereby details on each respondent’s marriage (beginning with the first) were recorded. With this information, we could determine the year the first marriage took place, the duration of marriage, and the duration between marriages or since the dissolution of the previous marriage. Ultimately, we summed the time spent between and after marriages (combining years spent divorced and widowed) and divided these person-years lived outside marriage by the number of years that had passed since first marriage. We multiplied this ratio by 100 (to produce a percentage value). Given high remarriage rates and short durations between dissolutions and higher-order marriages (Bracher, Santow, and Watkins 2003; Reniers 2003), an increase in even several percentage points of life spent outside of marriage is substantively meaningful (e.g., 1.5 more years out of 30 years since first becoming married corresponds to a 5-percentage-point-higher fraction of adult life spent married). This variable effectively captures variation in the portion of individuals’ lives spent outside of marriage and provides an additional marital dissolution dimension to test in conjunction with having experienced a marital dissolution and one’s number of dissolutions.

Approximately 4% of eligible respondents appeared to be in polygamous or at least concurrent marriages (married to multiple spouses who do not know they are not the only spouse) primarily concentrated in the south (Balaka district). Because polygamous or concurrent marriages are distinct and marital transitions in polygamous marriages have distinct patterns compared to dissolutions of monogamous marriages, these values of exposure to divorce/widowhood ‒ and therefore women and men who are currently or were previously in polygamous marriages ‒ are excluded from this variable and our analyses.

### 5.3 Regression choices

Our fixed-effects regressions also control for time-varying observable characteristics within respondents over the three waves. These regressions subsume time-invariant unobserved heterogeneity stemming from fixed individual, or contextual, factors in the individual fixed-effects parameter. This aspect is important since unobserved factors that jointly influence health and marital transitions (such as fixed personality characteristics, fixed local socioeconomic contexts, persistent differences in local HIV prevalence, and local cultural/religious norms, such as those regarding alcohol use or gender norms, potentially affecting both marriage and health) which might otherwise bias the analyses. Fixed-effect analyses also control for measurement error predating marital events prior to 2008 too (e.g., measurement error in the age of first marriage, if prior to 2008, is differenced out). By controlling for time-invariant characteristics through fixed-effects analyses found in Equation 1 (full regression models; stepwise models are shown within analytic tables), our approach accounts for a considerable amount of health and marriage selectivity among older Malawians:

(1) $$SF12Mental{\;}_{it}=M_{it}\beta_{1}+X_{it}\beta_{2}+\alpha_{i}+\varepsilon_{it}$$

Changes in SF12Mental_it_, the outcome, are measured by the SF-12 mental health score of an individual, *i*, at a certain time, *t*; *M*_*it*_ is the set of time-varying marital dissolution predictors ‒ the multiple dimensions ‒ that we test in this paper. As noted above, these measures are ever divorced/widowed, number of marital dissolutions, and the percentage of one’s life spent divorced and/or widowed since first being married. To allow a discontinuity for one marital dissolution, we include a time-varying dummy variable for being divorced/widowed at least once, and each additional separation. The former captures the effect of having ever experienced and the latter captures the marginal effect of subsequent dissolutions. Consequently, a value of one is subtracted from the number of marital dissolutions one experienced (except for those who have not been divorced or widowed) to empirically adjust for this discontinuity. *X*_*it*_ represents time-varying control variables ‒ current marital status and overall household savings (ln) in Malawi kwacha (in 2010, for instance, 150 kwacha were equal to approximately one US dollar). Age is excluded from these equations, as it is functionally a fixed characteristic, along with other time-invariant factors in the term α_i_. Time-varying error is captured by *ε*_*it*_. *R*^*2*^ goodness of fit values are presented, as well, in two forms: (1) calculations that do not account for explanatory power derived from individual fixed-effects (via *xtreg* command in Stata SE 16.1, from which model standard errors also stem from); and (2) calculations that account for explanatory power derived from the ‘absorbed’ individual fixed-effects (via *areg* command in Stata SE 16.1).

Since we are also interested in potential moderating effects between exposure to marital dissolutions and health, we test whether the following two factors act as buffers ‒ via interactions with our marital dissolution predictors ‒ relevant to rural Malawi: (1) between-wave changes in overall savings (ln) and (2) fixed region effects (to proxy for kinship system and gendered inequalities within kinship systems).

### 5.4 HIV considerations and sensitivity analyses

There are widely known effects that the presence of an HIV-positive individual in a union has on the likelihood of divorce and/or widowhood. But HIV status is not an ideal time-varying characteristic to include from an empirical perspective in these fixed-effects models: (1) it can only change once (becoming HIV-positive), and (2) the MLSFH did not conduct HIV testing in all waves, and respondents who were eligible to be tested could refuse (although refusal rates have typically been less than 10%; see Kohler et al. 2015). Although we exclude HIV status in our models, we conducted additional analyses (available upon request) that exclude individuals who are HIV-positive and then individuals and/or their matched spouses who have ever been diagnosed as HIV-positive to address these concerns; the results are nearly identical to those in the main text.

## 6. Descriptive results

The average SF-12 mental health scores are marginally higher than the standard of 50, but are, nonetheless, distributed among rural Malawians as one would expect in HICs. Over half of the women, and slightly less than half of men, in our sample have been divorced or widowed at least once. By 2012, over 20% of women and men experienced multiple marital dissolutions. Also, by 2012, women spent an average of 13% of their lives outside of marriage since first becoming married (approximately six years for a 65-year-old who first married at 18 years old), while men averaged 7% (approximately three years for a 65-year-old who first married at 18 years old); the large standard deviations indicate wide variation in this key predictor among women and men our sample. For additional context, the average age at first marriage for women in our sample was 19.4 years (6.9 standard deviation) and 24.3 years for men (6.9 standard deviation).

To further convey this variation and complexity in marital experiences, 23% of our respondents aged 45 years and older had been married three or more times, 45% had been married only once, and 8% had been divorced or widowed three or more times by 2012. Approximately one-quarter (24.9%) of our respondents experienced a divorce, 23.4% experienced widowhood, and only 6.4% reported experiencing both. By 2012, one-quarter of our respondents spent 68% or more of their adult life married, while another quarter spent 48% or less in unions. More-detailed descriptive statistics of our outcomes, time-varying independent variables, and key time-invariant variables employed in interaction effects are found in Table 1. These differences in the marital life course of mature adults may potentially contribute to our understanding of their substantial health differences (Myroniuk 2017; Payne, Mkandawire, and Kohler 2013).

## 7. Multivariate results

For mature Malawian women (Table 2), each additional marital dissolution ‒ after the first ‒ is associated with up to a 4.25-point increase in SF-12 mental health scores (models 2, 4, and 6). This change is about half of a standard deviation difference in these scores. Although having ever experienced a divorce or becoming widowed is consistently negatively associated with changes in mental health, these effects do not appear to be different from zero. Further, changes in the percentage of one’s life spent outside of marriage since first becoming married, current marital status, and savings are minimally ‒ if at all ‒ associated with changes in mental health.

For mature Malawian men (Table 3), in contrast to the above findings for women, changes in the percentage of years that men have spent divorced and/or widowed since first becoming married are strongly and negatively associated with SF-12 mental health scores (models 3, 5, and 6). Nevertheless, changes in one’s number of marital dissolutions or experiencing a first divorce/becoming widowed for the first time are not associated with changes in mental health. The same goes for one’s current marital status. However, increases in household savings are linked to increases in SF-12 mental health scores, even if these effects are modest (models 1‒6).

Table 4 tests whether savings or region of residence (proxying for kinship systems) moderate the effects of changes in substantive main effects of the dimensions of marital dissolutions for women and men.

For mature women (Table 4, model 1), the main effects of changes in one’s number of marital dissolutions are still associated with increases in mental health. Savings do not moderate this relationship. Further, the main effects of changes in one’s number of marital dissolutions are nullified when testing whether living in Malawi’s southern region (where women could have health advantages given the predominant matrilineal kinship system) ‒ which does not influence changes in mental health either (Table 4, model 2).

For mature men (Table 4, model 3), the interaction between the percentage of one’s life spent outside of marriage since the onset of first marriage and savings is marginal. Based on post-regression estimates (not presented), these results indicate that savings increasingly moderate the relationship between this dimension of marital dissolutions and health as the percentage of one’s life outside of marriage reaches beyond 10%. As savings increase, changes in mental health have a substantially steeper slope for those who have spent 10% or 15% of their lives outside of marriage, compared to those who have spent smaller percentages of their lives outside of marriage. Still, changes in mental health among these individuals are small, even with substantial increases in savings. Finally, living in the southern region does not exacerbate any mental health disadvantages associated with changes in the percent of their lives that men spend outside of marriage (Table 4, model 4).

## 8. Discussion

After accounting for fixed individual characteristics and key time-varying factors, changes in the number of marital dissolutions one has experienced and lifetime exposure to being divorced and/or widowed are still modestly associated with changes in mental health ‒ albeit, in opposite directions ‒ for women and men, respectively. The discrete effect of becoming divorced or widowed for the first time is not linked to changes in mental health for women or men, which is in line with a lack of gendered variation immediately following union dissolutions in HICs (e.g., Blekesaune 2008; Strohschein et al. 2005).

For men, larger portions of one’s life spent unmarried could result in a type of role strain (e.g., Pearlin 1989; Pearlin 1999). Although the causal mechanisms are not clear, men might face new emotional stresses since they must take up new tasks that were previously under the domain of their wives in order to survive; findings in HICs have not yet identified this relationship between new tasks and such stressors due to time spent unmarried, but these negative effects in men for a discrete event of divorce or widowhood have been found to be amplified with age (Umberson, Wortman, and Kessler 1992). Household savings ‒ albeit an imperfect measure affected to some extent by types of marital dissolutions and shared wealth of spouses ‒ offer a small buffering effect, but changes in savings do not moderate this relationship more; in previously noted post-regression predictions, changes in mental health are small at best ‒ a couple of tenths of one standard deviation, given even dramatic increases in savings (8 points on a logarithmic scale). It is possible that men are not reaping the financial protective effects of marriage, as men have often been found to benefit from in HICs (e.g., Murray 2000; Rendall et al. 2011). However, scholars have not tested if the ‘marriage premium’ even exists in rural Malawi. Lastly, the interaction between the percentage of one’s life spent outside of marriage and region do not appear substantive; we expected a demonstrable disadvantage for men in the southern region because of the manner in which women can easily dismiss their husbands (Schatz 2005) and that men generally do not receive inheritance rights in this region where matrilineal marriage and inheritance dominates. This ought to add motivation to the need to test for marriage premium effects.

For Malawian women, the results seem counterintuitive: Increases in the number of marital dissolutions are associated with increases in mental health scores. Considering that this relationship is not moderated by residence in the Southern region ‒ which would be expected because of the financial benefits of matrilineal inheritance or more marital agency in general ‒ the remaining explanation lies in the selectivity of those who experience dissolutions. We believe that there are two plausible yet empirically indistinguishable explanations: (1) Enough women in the MLSFH have gotten out of ‘bad’ marriages ‒ either through divorce or widowhood ‒ which otherwise would have been detrimental to their mental health, and/or (2) those in good mental health are the ones able to remarry after a divorce or becoming widowed and thus have experienced multiple marriages. Conversely, there are undoubtedly women who have never left their marriage for a plethora of reasons, and their mental health could suffer; given the lack of change in marital status, their effects within our regressions would not be as obvious. Another explanation in this context is that divorce is a health-empowering mechanism for women, often to avoid real or perceived HIV infection (Reniers 2003; Reniers 2008; Smith and Watkins 2005; Watkins 2004). If this is the case among this sample of mature Malawians ‒ then it is likely that there would be no, or little, detrimental effect of marital dissolutions on women’s mental health. Nonetheless, unpacking these mechanisms is an important task that in-depth, qualitative research could address. Understanding why someone’s third divorce or second time becoming widowed could actually be a boon for one’s mental health requires detailed, first-person narratives; such research would be a monumental contribution to understanding the health selection mechanisms surrounding marital dissolutions.

## 9. Limitations

Our study has its empirical and conceptual limitations. First, while our fixed-effects estimation procedure eliminates time-invariant confounders, it is still possible that unobserved time-varying characteristics that simultaneously affect changes in health and marital status bias the analyses. We cannot claim a causal relationship between lifetime exposure to these multiple dimensions of marital dissolutions and mental health. Second, missing data could bias our regression estimates, although several sets of sensitivity analyses ‒ including a complete case analysis (see Appendix Table 1) and multiple imputation (results not presented, and only appropriate based on female missing data due to the percentage of missing observations) do not substantively change our conclusions. The vast majority of missing data stemmed from the interviewer prompting issues on the SF-12 items in 2008 fieldwork as noted in previous research (Myroniuk 2017). Third, there are reliability issues regarding retrospective marital histories among rural Malawian women and men (Chae 2016) even after we ameliorated this concern as much as possible by considering the 2010 wave as the ‘ground truth’ marital history for an individual and creating values for our marriage variables in 2008 and 2012 based on 2010 reporting. Fourth, polygamy and concurrent marriages remain large unknowns in the extent to which exposure to marital dissolutions is associated with mental health; it is not even clear how to measure such exposure, whether it is time spent without multiple spouses or time spent before replacing a spouse or something else, but this would be an area of valuable pursuits, not only in Malawi but wherever being married to multiple spouses is practiced.

## 10. Theoretical contributions

Our research from rural Malawi provides a type of litmus test for many HICs where marriage, remarriage, and dissolution rates are lower but quite consequential for mental health outcomes. Measuring time outside of marriage should be more strongly considered in such settings. Going forward, research testing the relationships between marriage, marital dissolutions, and mental health ought to also consider the order and timing of life events more explicitly via sequence analyses (Billari 2001); marital events rarely happen in a vacuum and the order and timing are gendered. In Malawi, scholars have identified how specific life course events relate to the likelihood of HIV infection (Boileau et al. 2009). Applying these methods in understanding the risks of noncommunicable diseases and other health conditions, like poor mental health, is a logical next step in this line of scholarship. In the end, our sample of mature Malawian adults still serves to proxy for the social and demographic lives of a very sizable population in rural low-income countries in SSA. These results can inform what will be increasingly important research on the relationship between marital dissolutions and mental health in other African nations as noncommunicable diseases play a continually more important role in older individuals’ lives.

## 11. Public health considerations

Malawi, like most of SSA, is aging. Those who live to their 45^th^ birthdays are likely to live into their 70s (Payne, Mkandawire, and Kohler 2013). Malawi’s population of those 60 years and older will also grow nearly sevenfold by 2060 (UN 2015), and assessing the relationship between social factors and noncommunicable diseases such as depression, anxiety, and other adverse mental health outcomes is increasingly important in understanding population health as the HIV/AIDS pandemic slowly recedes. Given that the Malawi Ministry of Health is aware of how few resources, including healthcare worker training, have been put toward addressing population mental health, the changing demographics and burden of disease should be seen by the Malawian government as an opportunity to invest in the longevity of its citizens.

## Figures and Tables

**Table 1:** Descriptive statistics for respondents in 2008, 2010, and 2012 (means, percentages, and standard deviations where appropriate)

	Women 2008	Women 2010	Women 2012	Men 2008	Men 2010	Men 2012
SF-12 Mental Health (0–100)	51.5 (9.6)	50.0 (10.6)	52.0 (10.3)	55.1 (8.4)	53.3 (9.0)	54.4 (9.1)
Age	53.8 (11.9)	55.8 (11.9)	57.8 (11.9)	55.7 (10.8)	57.7 (10.8)	59.7 (10.8)
Ever divorced/widowed	50.5	56.1	59.1	43.6	45.7	46.9
*# Marital dissolutions*						
0	49.6	43.9	40.9	56.4	54.3	53.1
1	33.9	34.5	35.5	26.6	26.7	26.0
2	12.2	14.9	15.5	11.6	12.2	13.6
3+	4.3	6.7	8.1	2.7	6.8	7.4
% Years outside marriage	10.5 (16.9)	11.4 (17.4)	13.2 (18.9)	6.8(15.7)	7.4 (16.1)	7.7 (16.5)
Currently married	72.0	68.8	64.2	96.7	94.5	95.4
Savings MWK (ln)	3.6 (2.7)	4.5 (3.3)	4.6 (3.2)	4.5 (3.4)	5.3 (3.6)	5.5 (3.8)
Southern region	39.0	—	—	32.2	—	—

N (max)		670			516	

**Table 2:** Mature women, fixed-effects regressions predicting changes in mental health, 2008, 2010, 2012

	(1)		(2)		(3)		(4)		(5)		(6)	
Ever	−2.66	[−6.48,1.17]					−2.92	[−6.69,.85]	−4.01	[−9.63,1.61]	−3.58	[−9.18,2.02]
Divorced/ widowed (ref. never)	(1.95)	p=.17					(1.92)	p=.13	(2.86)	p=.16	(2.85)	p=.21
# Marital			2.11	[−.02,4.24]			2.28	[.09,4.47]			4.25	[1.67,6.83]
Dissolutions			(1.09)	p=.52			(1.12)	p=.04			(1.31)	p=.001
% Years					.07	[−.11,.26]			.09	[−09,.27]	.03	[−.16,.22]
Outside marriage					(.09)	p=.44			(.09)	p=.32	(.09)	p=.75
Not married	−.16	[−3.24,2.92]	−1.58	[−4.36,1.20]	−.16	[−3.48,3.16]	−.72	[−3.82,2.37]	.90	[−3.05,4.85]	.27	[−3.64,4.19]
(ref. married)	(1.57)	p=.92	(1.42)	p=.27	(1.69)	p=.92	(1.58)	p=.65	(2.01)	p=.65	(2.00)	p=.89
Savings	.13	[−.06,.33]	.13	[−.06,.32]	.14	[−07,.35]	.13	[−.06,.33]	.14	[−07,.36]	.14	[−07,.35]
MWK (In)	(.10)	p=.17	(.10)	p=.17	(.11)	p=.19	(.10)	p=.17	(.11)	p=.18	(.11)	p=.19
Constant	52.11	[5.11,54.10]	50.47	[49.22,51.73]	50.21	[48.03,52.39]	51.77	[49.76,53.77]	51.88	[48.75,55.01]	51.43	[48.3,54.56]
	(1.01)	p<001	(.64)	p<001	(1.11)	p<.001	(1.02)	p<.001	(1.59)	p<001	(1.59)	p<.001

Observations	1,844		1,844		1,464		1,844		1,464		1,464	
R^2^ Calculations: *Without fixed-effects*	.011		.006		.001		.008		.0001		.0003	
*With fixed-effects*	.461		.461		.446		.462		.447		.450	

*Notes:* 95% confidence intervals presented in brackets and continuous p-values reported. Standard errors, adjusted for individual clustering, in parentheses. Point estimates are the other values presented.

**Table 3:** Mature men, fixed-effects regressions predicting changes in mental health, 2008, 2010, 2012

	(1)		(2)		(3)		(4)		(5)		(6)	
Ever Divorced/widowed (ref. never)	−2.78 (2.87)	[−8.42,2.86] p=.33					−2.80 (2.87)	[−8.44,2.83] p=.33	−2.15 (3.20)	[−8.45,4.15] p=.50	−2.13 (3.24)	[−8.49,4.23] p=.51
# Marital Dissolutions			−.35 (1.53)	[−3.37,2.66] p=.82			−.41 (1.53)	[−3.42,2.59] p=.79			.84 (1.41)	[−1.93,3.61] p=.55
% Years Outside marriage					−.25 (.10)	[−.45,−.06] p=.01			−.25 (.10)	[−.45,−.05] p=.01	−.26 (.11)	[−.47,−. 05] p=.02
Not married (ref. married)	−.29 (2.44)	[−5.08,4.49] p=.90	−.71 (2.45)	[−5.52,4.10] p=.77	.92 (2.62)	[−4.22,6.06] p=.73	−.18 (2.48)	[−5.05,4.70] p=.94	1.22 (2.64)	[−3.97,6.40] p=.65	1.09 (2.67)	[−4.15,6.34] p=.68
Savings MWK (In)	.17 (.08)	[.01,. 33] p=.04	.16 (.08)	[−.00,.32] p=.05	.19 (.08)	[02,.35] p=.03	.17 (.08)	[.01,. 33] p=.04	.19 (.08)	[.03,.36] p=.02	.19 (.08)	[−.03,.36] p=.02
Constant	54.59 (1.32)	[52.01,57.17] p<001	53.49 (.65)	[52.21,54.77] p<001	55.10 (.76)	[53.60,56.60] p<.001	54.74 (1.44)	[51.90,57.57] p<001	55.98 (1.56)	[52.92,59.04] p<001	55.78 (1.62)	[52.59,58.97] p<.001

Observations R^2^ Calculations:	1,405		1,405		1,311		1,405		1,311		1,311	
*Without fixed- effects*	.007		.010		.007		.007		.008		.006	
*With fixed- effects*	.472		.471		.472		.472		.473		.473	

*Notes:* 95% confidence intervals presented in brackets and continuous p-values reported. Standard errors, adjusted for individual clustering, in parentheses. Point estimates are the other values presented.

**Table 4:** Mature women and men fixed-effects regressions, with key main effects and interactions, predicting changes in SF-12 mental health, 2008, 2010, 2012

	Women		Women		Men		Men	
	(1)		(2)		(3)		(4)	
# Marital dissolutions	4.59 (1.59)	[1.45,7.71] p = .00	2.88 (2.11)	[−1.26,7.02] p = .17				
Savings MWK (ln)	.16 (.11)	[−.07,.38] p = .17	.14 (.11)	[−.07,.35] p = .19				
# Marital dissolutions × Savings MWK (ln)	−.07 (.20)	[−.45,.32] p = .74						
# Marital dissolutions × Southern region			2.36 (2.57)	[−2.70,7.42] p = .36				
% Years outside marriage					−.32 (.12)	[−.56,−.07] p = .01	−.31 (.12)	[−.54,−.09] p = .01
Savings MWK (ln)					.09 (.09)	[−.09,.27] p = .31	.19 (.08)	[.03,.36] p = .02
% Years outside marriage × Savings MWK (ln)					.01 (.01)	[−.00,.02] p = .05		
% Years outside marriage × Southern region							.24 (.18)	[−.11,.60] p = .18

Observations R^2^ Calculations:	1,464		1,464		1,311		1,311	
*Without fixed-effects*	.0002		.0000		.007		.004	
*With fixed-effects*	.450		.451		.476		.473	

*Notes*: 95% confidence intervals presented in brackets and continuous p-values reported. Standard errors, adjusted for individual clustering, in parentheses. Point estimates are the other values presented. Other time-varying predictors in full expressions of models in Tables 2 and 3 (model 6) are included, but not presented, in these regressions.

## Footnotes

4 There are some differences, on key indicators across multiple waves of data, between those who have missing values for age at first marriage and those who do not. These differences do not appear to be substantive. For women with missing ages at first marriage, there is at most a 0.3 standard deviation difference in mental health from those whose age is not missing in the 2008 and 2012 waves; there is not an identifiable reason why this would be the case. In each wave, women with missing values for age at first marriage have experienced roughly 0.2 more marital dissolutions than those without these missing values. About 20% more women and men who live in the southern region of Malawi have missing values on age at first marriage than those who live elsewhere; this could be, in some way, due to matrilineal marriage systems, but the exact mechanism is not readily apparent.

5 The SF-12 was modified for the rural Malawian context (see Appendix Table A-2). Self-rated health questions pertaining to an individual’s general health, physical limitations, and emotional well-being are scored and weighted to derive a mental health score. A different weighting and scoring scheme are applied for a physical health score (not employed here).

6 These 64 cases are likely a result of polygamous or concurrent marriages, and they may only marginally bias our estimates due to their exclusion from the analyses. Only 2% more women are excluded, on these criteria, than men. There is only about 0.07^th^ of a standard deviation lower average SF-12 mental health scores among the excluded, as well ‒ nearly a substantively meaningless difference. As one could reasonably expect, the lowest percentage of these excluded individuals are found in the northern

## Appendix

**Table A-1:** Mature women fixed-effects regressions predicting changes in mental health, 2008, 2010, 2012, identical observations

	(1)		(2)		(3)		(4)		(5)		(6)	
Ever Divorced/ widowed (ref. never)	−3.80 (2.87)	[−9.43,1.83] p=.19					−3.50 (2.85)	[−9.11,2.11] p=.22	−4.01 (2.86)	[−9.63,1.61] p=.16	−3.58 (2.85)	[−9.18,2.02] p=.21
# Marital dissolutions			4.49 (1.25)	[2.04,6.94] p<001			4.37 (1.25)	[1.90,6.83] p=.001			4.25 (1.31)	1.67,6.83 p=.001
% Years Outside marriage					0.07 (0.09)	[−.11,.26] p=.44			0.09 (0.09)	[−09,.27] p=.32	0.03 (0.09)	[−.16,.22] p=.75
Not Married (ref. married)	1.24 (1.94)	[−2.58,5.06] p=.52	−0.66 (1.58)	[−3.76,2.45] p=.68	−0.16 (1.69)	[−3.48,3.16] p=.92	0.37 (1.94)	[−3.44,4.18] p=.85	0.90 (2.01)	[−3.05,4.85] p=.65	0.27 (2.00)	[−3.64,4.19] p=.89
Savings MWK (In)	0.15 (0.11)	[−07,.53] p=.17	0.14 (0.11)	[−07,.35] p=.20	0.14 (0.11)	[−07,.35] p=.19	0.14 (0.11)	[−07,.36] p=.19	0.14 (0.11)	[−07,.36] p=.18	0.14 (0.11)	[−07,.35] p=.19
Constant	52.70 (1.34)	[50.07,55.33] p<001	50.03 (0.70)	[48.66,51.40] p<001	50.21 (1.11)	[48.03,52.39] p<001	51.68 (1.37)	[48.98,54.37] p<.001	51.88 (1.59)	[48.75,55.01] p<001	51.43 (1.59)	[48.30,54.56] p<.001

Observations R^2^ Calculations:	1,464		1,464		1,464		1,464		1,464		1,464	
*Without fixed- effects*	.005		.0000		.001		.001		.0001		.0003	
*With fixed- effects*	.447		.449		.446		.450		.447		.450	

*Notes:* 95% confidence intervals presented in brackets and continuous p-values reported. Standard errors, adjusted for individual clustering, in parentheses. Point estimates are the other values presented.

**Table A-2:** SF-12 instrument comparison

Item #	SF-12 Standard (as found in Ware, Keller, and Kosinski 1995)	SF-12 Standard, in English, adapted for MLSFH and MLSFH-MAC but translated into chiChewa, chiTumbuka, and chiYao for data collection
1	In general, would you say your health is… *[Excellent; Very Good; Good; Fair; Poor]*	In general, would you say your health is… *[Much Better; Very Good; Good; Fair; Poor]*
2	The following items are about activities you might do during a typical day. Does your health now limit you in these activities? If so, how much? Moderate activities, such as moving a table, pushing a vacuum cleaner, bowling, or playing golf… *[Yes, Limited a Lot; Yes, Limited a Little; No, Not Limited at All]*	Do you have any health problems that limit you in carrying out moderate activities? For example, cooking and cleaning, walking to meetings in the village, or tending to cattle and livestock. If so, how much? *[Yes, Limited a Lot; Yes, Limited a Little/Moderately; No, Not Limited at All]*
3	The following items are about activities you might do during a typical day. Does your health now limit you in these activities? If so, how much? Climbing a flight of stairs… *[Yes, Limited a Lot; Yes, Limited a Little; No, Not Limited at All]*	Do you have any health problems that limit you in carrying out strenuous activities that you might do during a typical day, such as walking up a hill or walking up stairs? *[Yes, Limited a Lot; Yes, Limited a Little/Moderately; No, Not Limited at All]*
4	During the past 4 weeks, have you had any of the following problems with your work or regular daily activities as a result of your physical health? Accomplished less than you would like... *[Yes; No]*	During the past 4 weeks, have you accomplished less than you would like, as a result of your physical health? *[Yes; No]*
5	During the past 4 weeks, have you had any of the following problems with your work or regular daily activities as a result of your physical health? Were limited in the kind of work or other activities… *[Yes; No]*	During the past 4 weeks, have you been unable to do certain things as a result of your physical health? *[Yes; No]*
6	During the past 4 weeks, have you had any of the following problems with your work or regular daily activities as a result of any emotional problems (such as feeling depressed or anxious)? Accomplished less than you would like... *[Yes; No]*	During the past 4 weeks, have you accomplished less than you would like, as a result of any emotional problems (such as feeling depressed or anxious)? *[Yes; No]*
7	During the past 4 weeks, have you had any of the following problems with your work or regular daily activities as a result of any emotional problems (such as feeling depressed or anxious)? Didn’t do work or other activities as carefully as usual... *[Yes; No]*	During the past 4 weeks, did you do your work or other activities less carefully than usual, as a result of any emotional problems (such as feeling depressed or anxious)? *[Yes; No]*
8	During the past 4 weeks, how much did pain interfere with your normal work (including both work outside the home and housework)? *[Not at All; A Little Bit; Moderately; Quite a Bit; Extremely]*	During the past 4 weeks, how much did pain interfere with your normal work (including both work outside the home and housework)? *[Not at All; A Little Bit; Moderately; Quite a Bit; Extremely]*
9	These questions are about how you feel and how things have been with you during the past 4 weeks. For each question, please give the one answer that comes the closest to the way you have been feeling. How much of the time during the past 4 weeksHave you felt calm and peaceful? *[All of the Time; Most of the Time; A Good Bit of the Time; Some of the Time; A Little of the Time; None of the Time]*	How much of the time during the past 4 weeks have you felt calm and peaceful? *[All of the Time; Most of the Time; Some of the Time; A Little of the Time; None of the Time]*
10	These questions are about how you feel and how things have been with you during the past 4 weeks. For each question, please give the one answer that comes the closest to the way you have been feeling. How much of the time during the past 4 weeksDid you have a lot of energy? *[All of the Time; Most of the Time; A Good Bit of the Time; Some of the Time; A Little of the Time; None of the Time]*	How much of the time during the past 4 weeks did you have a lot of energy? *[All of the Time; Most of the Time; Some of the Time; A Little of the Time; None of the Time]*
11	These questions are about how you feel and how things have been with you during the past 4 weeks. For each question, please give the one answer that comes the closest to the way you have been feeling. How much of the time during the past 4 weeksHave you felt downhearted and blue? *[All of the Time; Most of the Time; A Good Bit of the Time; Some of the Time; A Little of the Time; None of the Time]*	How much of the time during the past 4 weeks have you felt downhearted and depressed? *[All of the Time; Most of the Time; Some of the Time; A Little of the Time; None of the Time]*
12	During the past 4 weeks, how much of the time has your physical health or emotional problems interfered with your social activities (like visiting with friends, relatives, etc.)? *[All of the Time; Most of the Time; Some of the Time; A Little of the Time; None of the Time]*	During the past 4 weeks, how much of the time has your physical health or emotional problems interfered with your social activities (like visiting with friends, relatives, etc.)? *[All of the Time; Most of the Time; Some of the Time; A Little of the Time; None of the Time]*
